# Diagnosis and misdiagnosis of necrotizing soft tissue infections: three case reports

**DOI:** 10.1186/1757-1626-1-252

**Published:** 2008-10-20

**Authors:** Engelbert Schröpfer, Stephan Rauthe, Thomas Meyer

**Affiliations:** 1 Department of General-, Visceral-, Vascular- and Transplant-Surgery, Julius-Maximilians-University of Würzburg, Würzburg, Germany; 2 Department of Pathology, Julius-Maximilians-University of Würzburg, Würzburg, Germany

## Abstract

**Background:**

Today, gas gangrene is rare, but still many of the patients die, despite having received timely treatment.

**Case presentation:**

This report highlights the cases of three different patients, who were transferred to our surgical department in 2006. The first patient (Patient_A), with the suspected diagnosis "femoral hematoma", a second patient (Patient_B) because of an "acute abdomen" and the third patient (Patient_C) with suspected gas gangrene of the right leg.

**Conclusion:**

The first two cases demonstrate gas gangrene should always be kept in mind, especially in high-risk-patients. Though, the third case shows that severe consequences because of a precipitate diagnosis can be avoided by careful evaluation.

## Background

Anaerobic infections by exotoxin-producing Clostridium perfringens are rare [1]. Due to systemic progression of the infection gas gangrene still remains life-threatening when detected late. We report on three cases of necrotizing soft tissue infections (NSTI). The first two patients suffering from NSTI were admitted to our hospital with different provisional diagnoses, one with compartment syndrome, the second with appendicitis. Despite immediate treatment both patients died within 24 hours after admittance to our hospital. The third patient was free of NSTI, although evincing all typical symptoms of gas gangrene.

## Case presentation

### First case

**Figure 1:**
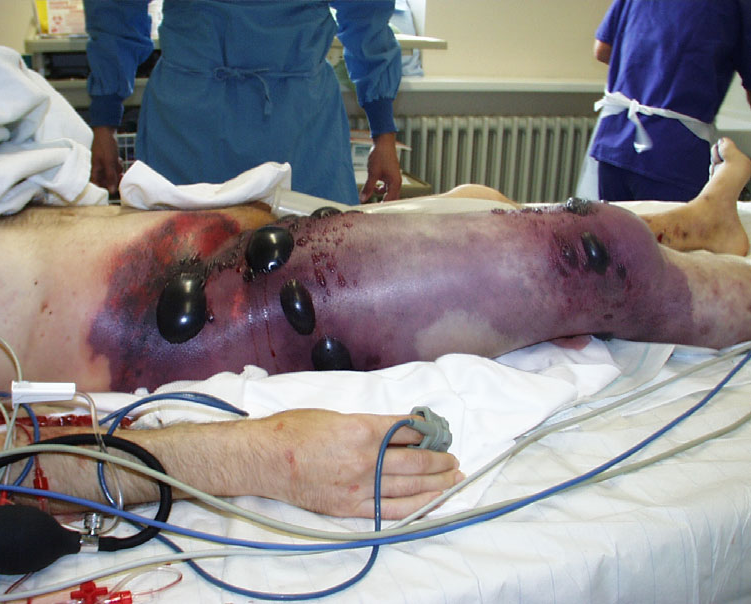
**Preoperative photography of the first patient**. First patient on the intensive ward before hemipelvectomy: The right thigh is swollen, edemous and discoloured. An impressive crepitus is already palpable. At this juncture the patient is in shock.

**Figure 2:**
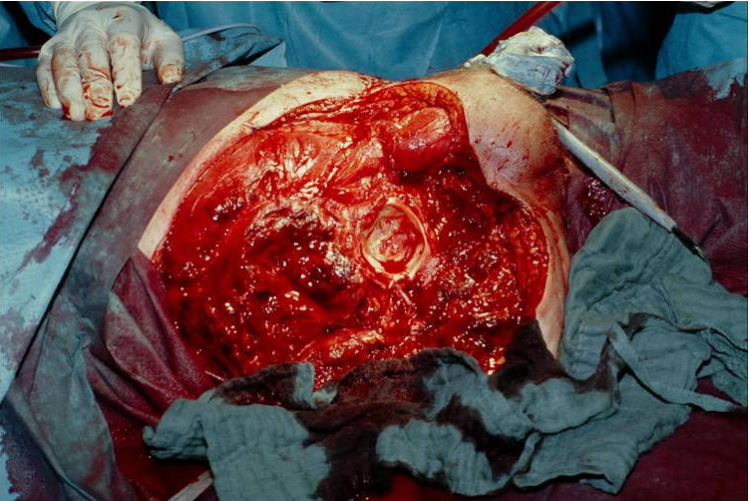
**Photography during operation after hemipelvectomy on the right side with view of the acetabulum**. View on the acetabulum and the pelvic muscles after hemipelvectomy was performed. Necrosis has already spread into the psoas muscle, therefore surgical resection of the necrotic tissue is not possible any more. Afterwards bandage of the open wound is performed and the patient is transmitted to intensive care unit for further treatment.

The first case reports on a 47-year-old man suffering from myelodysplastic syndrome for several years. Furthermore, he had inguinal and scrotal ulcerations, refractory to all previous treatments since 2002. Before admission to our university hospital with the suspected diagnosis of compartment syndrome, he was first admitted to a smaller hospital with a deep venous thrombosis of the right leg. Upon arrival in our hospital the patient was already in shock. On examination his right leg was edematous and discoloured with crepitus on deep palpation (Fig. 1). According to the accompanying documents these symptoms had existed for two days. Due to the provisional diagnosis of NSTI the patient was immediately taken to the operating room after initial cardiovascular stabilization in our intensive care unit. Broad-spectrum antibiotics were administered prior to operation. After hemipelvectomy (Fig. 2) a massive spreading of the NSTI, including necrosis of the psoas muscle, was detectable. Therefore no further surgical debulking of the infected tissue was possible. Despite intensive therapy the patient died eight hours after admission to our hospital.

**Figure 3:**
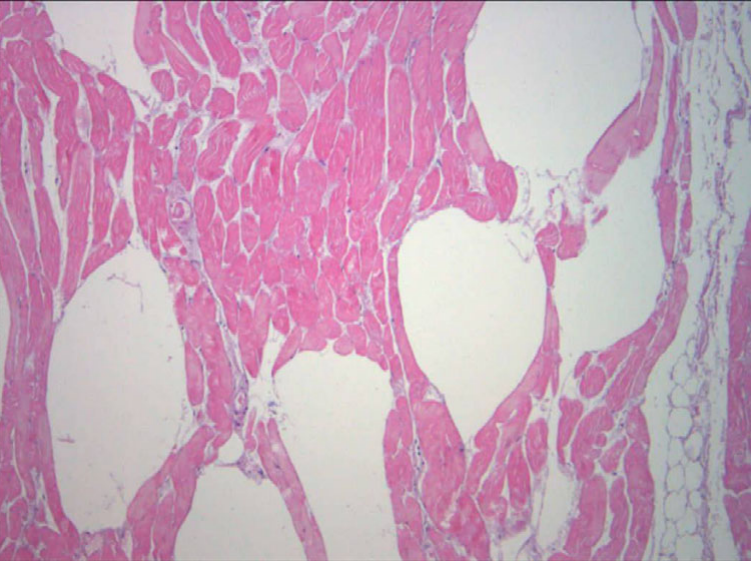
**Pathologic findings of the muscle tissue**. Postoperative pathologic findings of the muscle tissue obtained during surgical removal. Hämatoxylin-Eosin-stain, zoom 100×. Infected tissue with gas-inclusion between the muscle-fibres can be seen.

**Figure 4:**
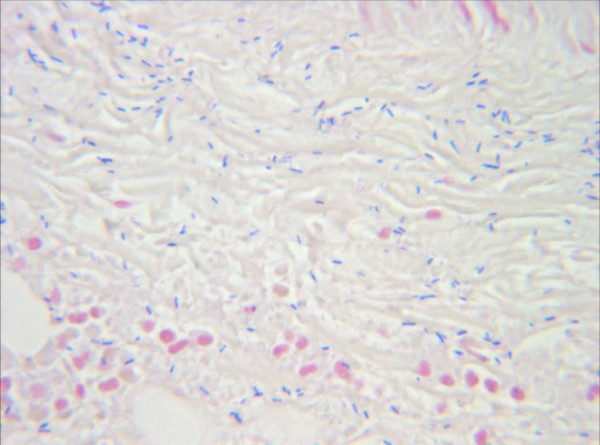
**Pathologic findings of the muscle tissue**. Postoperative pathologic findings of the muscle tissue obtained during surgical removal. Gram-stain, Zoom 1000×. Identification of gram-positive, rod-shaped, anaerobic, spore-forming bacteria in the infected muscle tissue. The result is highly compatible to an infection with clostridium perfringens.

The provisional diagnosis of gas gangrene caused by clostridium perfringens was confirmed by microbiological and pathological findings of the tissue obtained during surgery (Fig. 3, Fig. 4).

### Second case

**Figure 5:**
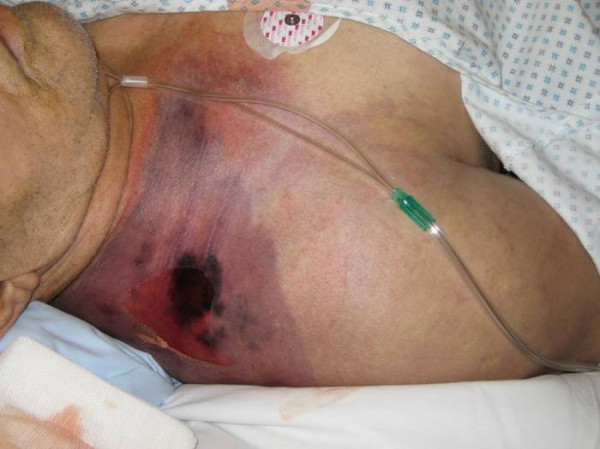
**Preoperative photography of the second patient**. Erythema of the right shoulder of patient two before operation. At this juncture crepitus is palpable in the subcutaneous tissue of the shoulder and the upper arm. In comparison, the erythema shows a massive progression

The second patient, a 73-year-old man suffering from nausea, emesis and diarrhoea was admitted to our hospital for further treatment with the diagnosis acute appendicitis. Besides abdominal pain, a physical examination demonstrated haematoma of the right shoulder. Computer tomography of the abdomen revealed a cecal tumor with hepatic and splenic metastasis. In addition, a progression of the haematoma with crepitus on palpation appeared (Fig. 5). A provisional diagnosis of NSTI was made and the patient was immediately taken to the operation room for forequarter amputation of the right arm. Due to progression of necrosis, nearly all pectoral, spinal and scapular muscles were resected. Although the perioperative antibiotic treatment with penicillin was intensified by the addition of clindamycin and ceftriaxone in the intensive care unit, a further progression of necrosis could be observed. The patient died in the intensive care unit 14 hours after admission. Pathologic findings confirmed the diagnosis clostridial infection.

### Third case

**Figure 6:**
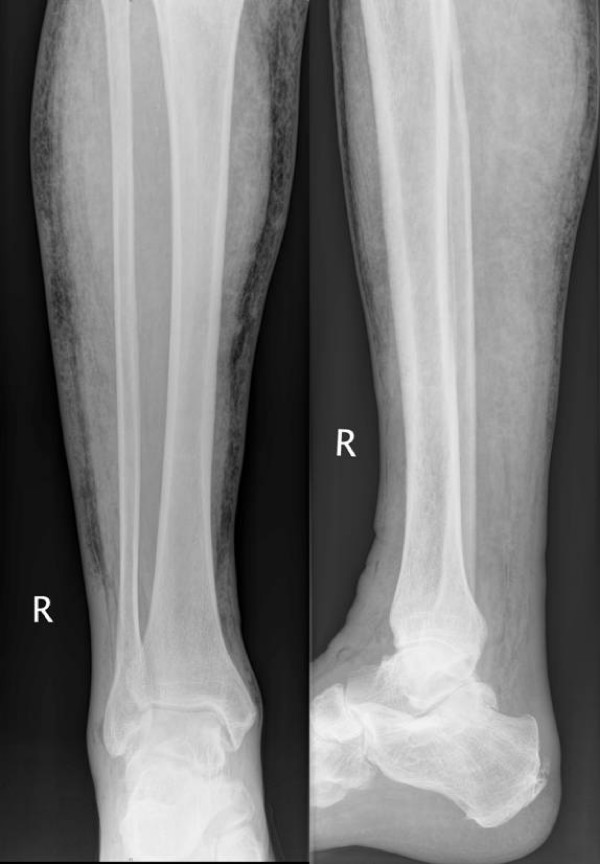
**X-Ray Images of the right lower leg of patient three**. X-Ray Images in the anteroposterior and lateral projection of the right lower leg and ankle joint of the third patient: Free gas in the subcutaneous and in the muscle tissue can be seen. According to these images, the surgeon in charge of another hospital admitted the patient to our department with the provisional diagnosis "gas gangrene of the right leg" for amputation and intensive care monitoring.

The third case reports on a 68-year-old patient, admitted to our department because of "gas gangrene of the right leg". Medical history revealed leucocytoclastic vasculitis and diabetic foot ulcer on the right side sine six months. Two days before debridement of the ulcer was performed by a local GP. Physical examination demonstrated crepitation of the lower right leg and right thigh. Inflammatory parameters in laboratory findings were slightly increased (leucocytes 11.600/μl [5.000–10.000/μl], CRP 2.52 mg/dl [0–0.5 mg/dl]). X-ray imaging revealed intramuscular and subcutaneous gas (Fig. 6). The patient was immediately taken to the operation room for superficial skin incision of the medial lower leg. Both, the subcutaneous and the muscle tissue were raised by gas. The entire tissue appeared healthy and no necrosis could be observed. Biopsy of the muscle was taken and immediately sent to the microbiology department. Fasciotomy of the medial and lateral lower leg and the lateral thigh was performed. Microbiologic findings of the muscle tissue revealed no bacterial growth. The patient was admitted to our intensive care unit for antibiotic treatment and hourly wound monitoring. Further observation showed healthy muscle tissue and normal white blood cell count. Therefore we performed secondary suture of the fasciotomy wounds and surgical treatment of the foot ulcer. The patient was discharged from the intensive care unit three days after admission.

## Discussion

Clostridium perfringens is an aerotolerant spore-forming, gram-positive bacterium found in soil and the intestinal tract of humans and other vertebrates [2,3]. Proliferation of Clostridium perfringens requires changing normal environmental conditions to an anaerobic milieu. Such conditions can be caused by earlier widespread destruction of soft tissue, yet clostridial gas gangrene can also be found without a prior trauma [4]. Different risk factors, e.g. diabetes, malignancy, immunodeficiency and vascular diseases are known. Due to the growing number of diabetics and the elderly, NSTI, especially in absence of trauma, may become more important in the future.

All three patients suffered from at least one risk factor, none of them remembering a recent trauma. However, possible entry points for bacteria can be identified in patient one and three. In case one and two NSTI should have been considered as a differential diagnosis from the beginning. Due to the association of NSTI with large and contaminated wounds, the wrong diagnosis was made initially. As surgical sanitation of the infection is essential in NSTI, early diagnosis is the most important factor for survival [5].

A review of the English literature by Lanting et al. revealed three cases of spontaneous gas gangrene of the shoulder [6]. The primary diseases in these cases were diabetes with peptic ulcer disease, diabetes and ischemic heart disease and radiation colitis [7-9]. A strong relation between gastrointestinal malignancies and NSTI was shown by Kornbluth et al. [10], therefore colonoscopy is recommended for all patients with spontaneous clostridium infection [11].

The third case demonstrates the necessity of comparing radiological, clinical and intraoperative findings. According to literature, 79.2% of the patients with NSTI were in shock and all patients were suffering from enormous pain at the wound site [12]. Patients with a white blood-cell-count ≤ 15.400/μl have only a 1% chance of developing NSTI [13]. The number of white blood-cells in the third patient was slightly increased; no pain or signs of shock could be observed. In order to be able to inspect the muscle tissue, we decided on surgical treatment. After incision, muscle tissue could be observed and a biopsy was taken. Due to microbiological findings clostridial infection could be excluded. Detection of free gas in the tissue is not an indisputable indicator for bacterial infection. Therefore, a precise comparison between clinical and radiological findings is required before a decision on radical surgical treatment is made. Through fasciotomy a sufficient wound control in the first days after admission was possible.

## Conclusion

In patients suffering from NSTI, early diagnosis is essential for sufficient treatment and survival [14]. Although NSTI is well known, it was not considered in the first two cases due to absence of trauma. Despite all efforts, both patients died because surgical resection of the necrotic tissue was not possible. Nether less, free gas in the tissue and crepitus are not necessarily caused by NSTI and the diagnosis is not always straightforward [15].

## Competing interests

The authors declare that they have no competing interests.

## Authors' contributions

ES analyzed the data and drafted the manuscript and was the main coordinator. SR carried out the immunohistochemical staining. TM participated in editorial support.

## Consent

Written consent for publication was obtained from the relatives in case 1 and 2 and from the patient in case 3
